# Supplementary material for: Local nuclear to cytoplasmic ratio regulates H3.3 incorporation via cell cycle state during zygotic genome activation
Source: EMBO Rep. 2025 Nov 11;26(23):5703–27. doi: 10.1038/s44319-025-00596-1 (PMC12678481; doi:10.1038/s44319-025-00596-1)
Supplement: Supplementary file 1 — Appendix [file 44319_2025_596_MOESM1_ESM.pdf]

Appendix

Local nuclear to cytoplasmic ratio regulates H3.3 incorporation via cell cycle state during zygotic genome activation

Anusha D. Bhatt[1], Madeleine G. Brown[1], Aurora B. Wackford[1], Yuki Shindo[1], Amanda A. Amodeo[1,2,*]

[1]Department of Biological sciences, Dartmouth College, Hanover, NH 03755, USA

[2] Lead contact

*Correspondence: amanda.amodeo@dartmouth.edu

Table of Contents

Table	Page number
Appendix Table S1: Statistics for Figure EV1C	3
Appendix Table S2: Statistics for Figure 2B	3
Appendix Table S3: Statistics for Figure EV1E	3
Appendix Table S4: Statistics for Figure EV1G	4
Appendix Table S5: Statistics for Figure EV1H	4
Appendix Table S6: Statistics for Figure 3B	4
Appendix Table S7: Statistics for Figure 3C	6
Appendix Table S8: Statistics for Figure EV2G	7
Appendix Table S9: Statistics for Figure EV2I	8
Appendix Table S10: Statistics for Figure EV4B	9

Appendix Table S1: 2-way ANOVA results of H3-Dendra2 and H3.3-Dendra2 on chromatin over NC10 - NC13 as shown in Figure EV1C.

Comparison groups	Difference in means	Adjusted p-value
NC11_H3 - NC10_H3	-0.0627471	0.98843294
NC12_H3 - NC11_H3	-0.097712	0.88298162
NC13_H3 - NC12_H3	-0.220603	0.08782752
NC11_H3.3 - NC10_H3.3	0.37315126	0.00040692
NC12_H3.3 - NC11_H3.3	0.39265816	0.00019202
NC13_H3.3 - NC12_H3.3	0.37344713	0.00040232

Appendix Table S2: 2-way ANOVA results of H3-Dendra2 and H3.3-Dendra2 nuclear concentrations in the interphase nucleus during NC10 - NC13 as shown in Figure 2B.

Comparison groups	Difference in means	Adjusted p-value
NC11_H3 - NC10_H3	-0.1408876	0.18475814
NC12_H3 - NC11_H3	-0.1115503	0.44067226
NC13_H3 - NC12_H3	-0.1364362	0.21426967
NC11_H3.3 - NC10_H3.3	-0.0241768	0.99871742
NC12_H3.3 - NC11_H3.3	-0.077933	0.56567533
NC13_H3.3 - NC12_H3.3	-0.0730441	0.63966091

Appendix Table S3: 2-way ANOVA results of initial H3-Dendra2 and H3.3-Dendra2 import rates during NC11 - NC13 as shown in Figure EV1E.

Comparison groups	Difference in means	Adjusted p-value
NC12_H3 - NC11_H3	-0.381055416	0.00016788
NC12_H3.3 - NC11_H3.3	-0.303255393	0.00010136
NC13_H3 - NC12_H3	-0.34119875	0.00110214
NC13_H3.3 - NC12_H3.3	-0.356357728	3.46E-06
NC12_H3.3 - NC12_H3	0.077800023	0.99461495
NC13_H3.3 - NC13_H3	0.062641045	0.99943292

Appendix Table S4: 2-way ANOVA results for the different regions in the parallel embryo experiment for photobleaching correction for chromatin (NC10 vs NC13) as shown in Figure EV1G.

Comparison groups	Difference in means	Adjusted p-value
NC10_Outside - NC10_Image area	-0.015039	0.99820702
NC10_Parallel embryo - NC10_Image area	-0.006684	0.99993379
NC10_Parallel embryo - NC10_Outside	0.00835498	0.99985763
NC13_Outside - NC13_Image area	0.05703447	2.35E-06
NC13_Parallel embryo - NC13_Image area	0.05682858	2.58E-06
NC13_Parallel embryo - NC13_Outside	-0.0002059	1
NC13_Image area - NC10_Image area	-0.6050886	0
NC13_Outside - NC10_Outside	-0.5330152	0
NC13_Parallel embryo - NC10_Parallel embryo	-0.5415761	0

Appendix Table S5: 2-way ANOVA results for the different regions in the parallel embryo experiment for photobleaching correction for interphase nuclear concentration (NC10 vs NC13) as shown in Figure EV1H.

Comparison groups	Difference in means	Adjusted p-value
NC10_Outside - NC10_Image area	-0.0030836	0.99990859
NC10_Parallel embryo - NC10_Image area	-0.0011564	0.99999823
NC10_Parallel embryo - NC10_Outside	0.00192728	0.99998957
NC13_Outside - NC13_Image area	-0.004531	0.6781679
NC13_Parallel embryo - NC13_Image area	0.02075448	1.31E-11
NC13_Parallel embryo - NC13_Outside	0.02528553	1.73E-12
NC13_Image area - NC10_Image area	-0.3123329	9.00E-14
NC13_Outside - NC10_Outside	-0.3137803	9.00E-14
NC13_Parallel embryo - NC10_Parallel embryo	-0.2904221	9.00E-14

Appendix Table S6: 2-way ANOVA results of chimeras compared to H3-Dendra2 and H3.3-Dendra2 on chromatin over NC10 - NC13 as shown in Figure 3B.

Comparison groups	Difference in means	Adjusted p-value
NC10_H3 - NC10_ASVM	-1.55E-15	1
NC10_H3.3 - NC10_ASVM	3.33E-16	1
NC10_S31A - NC10_ASVM	0	1

NC10_SVM - NC10_ASVM	-1.11E-16	1
NC10_H3.3 - NC10_H3	1.89E-15	1
NC10_S31A - NC10_H3	1.55E-15	1
NC10_SVM - NC10_H3	1.44E-15	1
NC10_S31A - NC10_H3.3	-3.33E-16	1
NC10_SVM - NC10_H3.3	-4.44E-16	1
NC10_SVM - NC10_S31A	-1.11E-16	1
NC11_H3 - NC11_ASVM	-0.002548004	1
NC11_H3.3 - NC11_ASVM	0.43335039	2.05E-06
NC11_S31A - NC11_ASVM	0.195753042	0.32719825
NC11_SVM - NC11_ASVM	-0.055004006	0.99999902
NC11_H3.3 - NC11_H3	0.435898394	1.74E-06
NC11_S31A - NC11_H3	0.198301045	0.30496131
NC11_SVM - NC11_H3	-0.052456002	0.99999955
NC11_S31A - NC11_H3.3	-0.237597348	0.08064289
NC11_SVM - NC11_H3.3	-0.488354396	6.01E-08
NC11_SVM - NC11_S31A	-0.250757047	0.04722605
NC12_H3 - NC12_ASVM	0.007934945	1
NC12_H3.3 - NC12_ASVM	0.934203465	0
NC12_S31A - NC12_ASVM	0.527625335	4.57E-09
NC12_SVM - NC12_ASVM	-0.11622197	0.97313608
NC12_H3.3 - NC12_H3	0.92626852	0
NC12_S31A - NC12_H3	0.51969039	7.72E-09
NC12_SVM - NC12_H3	-0.124156915	0.95000571
NC12_S31A - NC12_H3.3	-0.40657813	1.08E-05
NC12_SVM - NC12_H3.3	-1.050425436	0
NC12_SVM - NC12_S31A	-0.643847306	0
NC13_H3 - NC13_ASVM	-0.038619561	1
NC13_H3.3 - NC13_ASVM	1.481699114	0
NC13_S31A - NC13_ASVM	0.861241303	0
NC13_SVM - NC13_ASVM	-0.139872287	0.8684167
NC13_H3.3 - NC13_H3	1.520318675	0
NC13_S31A - NC13_H3	0.899860864	0
NC13_SVM - NC13_H3	-0.101252726	0.99386895
NC13_S31A - NC13_H3.3	-0.620457811	0
NC13_SVM - NC13_H3.3	-1.621571401	0
NC13_SVM - NC13_S31A	-1.00111359	0

Appendix Table S7: 2-way ANOVA results for nuclear concentrations of chimeras compared to H3-Dendra2 and H3.3-Dendra2 in the interphase nucleus during NC10 - NC13 as shown in Figure 3C.

Comparison groups	Difference in Means	Adjusted p-value
NC10_H3 - NC10_ASVM	-0.0055188	1
NC10_H3.3 - NC10_ASVM	-0.0055188	1
NC10_S31A - NC10_ASVM	0.00028757	1
NC10_SVM - NC10_ASVM	-0.0055188	1
NC10_H3.3 - NC10_H3	7.94E-11	1
NC10_S31A - NC10_H3	0.00580633	1
NC10_SVM - NC10_H3	-1.06E-11	1
NC10_S31A - NC10_H3.3	0.00580633	1
NC10_SVM - NC10_H3.3	-9.00E-11	1
NC10_SVM - NC10_S31A	-0.0058063	1
NC11_H3 - NC11_ASVM	0.00619749	1
NC11_H3.3 - NC11_ASVM	0.12290828	0.17906869
NC11_S31A - NC11_ASVM	0.04818638	0.99931011
NC11_SVM - NC11_ASVM	0.00128513	1
NC11_H3.3 - NC11_H3	0.11671079	0.50685044
NC11_S31A - NC11_H3	0.0419889	0.99998836
NC11_SVM - NC11_H3	-0.0049124	1
NC11_S31A - NC11_H3.3	-0.0747219	0.91786474
NC11_SVM - NC11_H3.3	-0.1216232	0.19276663
NC11_SVM - NC11_S31A	-0.0469013	0.99952054
NC12_H3 - NC12_ASVM	0.0202021	1
NC12_H3.3 - NC12_ASVM	0.1705302	0.00518502
NC12_S31A - NC12_ASVM	0.0328656	0.99999765
NC12_SVM - NC12_ASVM	-0.0434382	0.99983517
NC12_H3.3 - NC12_H3	0.1503281	0.1149165
NC12_S31A - NC12_H3	0.0126635	1
NC12_SVM - NC12_H3	-0.0636403	0.99621661
NC12_S31A - NC12_H3.3	-0.1376646	0.06981896
NC12_SVM - NC12_H3.3	-0.2139684	8.38E-05
NC12_SVM - NC12_S31A	-0.0763038	0.90297737
NC13_H3 - NC13_ASVM	0.00965776	1
NC13_H3.3 - NC13_ASVM	0.22337804	3.21E-05
NC13_S31A - NC13_ASVM	0.06052475	0.9889634
NC13_SVM - NC13_ASVM	-0.0863012	0.77259947
NC13_H3.3 - NC13_H3	0.21372028	0.00139254

NC13_S31A - NC13_H3	0.05086699	0.99979886
NC13_SVM - NC13_H3	-0.095959	0.82075511
NC13_S31A - NC13_H3.3	-0.1628533	0.01001226
NC13_SVM - NC13_H3.3	-0.3096793	2.76E-09
NC13_SVM - NC13_S31A	-0.146826	0.03597147

Appendix Table S8: 2-way ANOVA results of initial import rates of chimeras compared to H3-Dendra2 and H3.3-Dendra2 during NC11 - NC13 as shown in Figure EV2G.

Comparison groups	Difference in Means	Adjusted p-value
NC12_ASVM - NC11_ASVM	-0.346793281	6.39E-06
NC12_H3 - NC11_H3	-0.381055416	0.00016788
NC12_H3.3 - NC11_H3.3	-0.303255393	0.00010136
NC12_S31A - NC11_S31A	-0.320997327	3.31E-05
NC12_SVM - NC11_SVM	-0.363066437	0.0003963
NC13_ASVM - NC12_ASVM	-0.398034537	2.36E-07
NC13_H3 - NC12_H3	-0.34119875	0.00110214
NC13_H3.3 - NC12_H3.3	-0.356357728	3.46E-06
NC13_S31A - NC12_S31A	-0.341763381	8.82E-06
NC13_SVM - NC12_SVM	-0.400466562	6.55E-05
NC13_SVM - NC13_ASVM	-0.018705181	1
NC13_SVM - NC13_H3	-0.041278832	0.99999904
NC12_ASVM - NC12_H3	0.034262135	0.99999964
NC12_H3.3 - NC12_ASVM	0.043537888	0.99995511
NC12_H3.3 - NC12_H3	0.077800023	0.99461495
NC12_H3.3 - NC12_S31A	0.017741934	1
NC12_H3.3 - NC12_SVM	0.059811044	0.99966109
NC12_S31A - NC12_ASVM	0.025795954	0.99999994
NC12_S31A - NC12_H3	0.060058089	0.99964501
NC12_S31A - NC12_SVM	0.04206911	0.99999497
NC12_SVM - NC12_ASVM	-0.016273156	1
NC12_SVM - NC12_H3	0.017988979	1
NC13_ASVM - NC13_H3	-0.022573651	1
NC13_H3.3 - NC13_ASVM	0.085214697	0.95767702
NC13_H3.3 - NC13_H3	0.062641045	0.99943292
NC13_H3.3 - NC13_S31A	0.003147587	1
NC13_H3.3 - NC13_SVM	0.103919878	0.93613851
NC13_S31A - NC13_ASVM	0.082067109	0.96862842
NC13_S31A - NC13_H3	0.059493458	0.99968081

NC13_S31A - NC13_SVM	0.100772291	0.94917367

Appendix Table S9: 2-way ANOVA results of hatch rates of chimeras compared to yw;; and H3.3-Dendra2 during a 5-day period as shown in Figure EV2I.

Comparison groups	Difference in Means	Adjusted p-value
H3.3:Day1 - y,w;;:Day1	4.2	0.99999998
ASVM:Day1 - y,w;;:Day1	-5.55	0.99999821
SVM:Day1 - y,w;;:Day1	-5.05	0.99999972
S31A:Day1 - y,w;;:Day1	-13.3	0.75626798
ASVM:Day1 - H3.3:Day1	-9.75	0.98605218
SVM:Day1 - H3.3:Day1	-9.25	0.99272729
S31A:Day1 - H3.3:Day1	-17.5	0.24028858
SVM:Day1 - ASVM:Day1	0.5	1
S31A:Day1 - ASVM:Day1	-7.75	0.99974046
S31A:Day1 - SVM:Day1	-8.25	0.99930744
H3.3:Day2 - y,w;;:Day2	6.2	0.9999601
ASVM:Day2 - y,w;;:Day2	-5.35	0.99999912
SVM:Day2 - y,w;;:Day2	-4.6	0.99999996
S31A:Day2 - y,w;;:Day2	-11.35	0.92985426
ASVM:Day2 - H3.3:Day2	-11.55	0.91772101
SVM:Day2 - H3.3:Day2	-10.8	0.9567487
S31A:Day2 - H3.3:Day2	-17.55	0.23569652
SVM:Day2 - ASVM:Day2	0.75	1
S31A:Day2 - ASVM:Day2	-6	0.99999711
S31A:Day2 - SVM:Day2	-6.75	0.99997499
H3.3:Day3 - y,w;;:Day3	8.2	0.99679111
ASVM:Day3 - y,w;;:Day3	-3.65	1
SVM:Day3 - y,w;;:Day3	0.35	1
S31A:Day3 - y,w;;:Day3	-14.4	0.61597803
ASVM:Day3 - H3.3:Day3	-11.85	0.8970404
SVM:Day3 - H3.3:Day3	-7.85	0.99927521
S31A:Day3 - H3.3:Day3	-22.6	0.01972928
SVM:Day3 - ASVM:Day3	4	1
S31A:Day3 - ASVM:Day3	-10.75	0.97654847
S31A:Day3 - SVM:Day3	-14.75	0.66988878
H3.3:Day4 - y,w;;:Day4	4.8	0.99999967
ASVM:Day4 - y,w;;:Day4	-2.7	1
SVM:Day4 - y,w;;:Day4	-0.7	1
S31A:Day4 - y,w;;:Day4	-13.7	0.70747093

ASVM:Day4 - H3.3:Day4	-7.5	0.99964337
SVM:Day4 - H3.3:Day4	-5.5	0.99999849
S31A:Day4 - H3.3:Day4	-18.5	0.15975931
SVM:Day4 - ASVM:Day4	2	1
S31A:Day4 - ASVM:Day4	-11	0.96981642
S31A:Day4 - SVM:Day4	-13	0.85742986
H3.3:Day5 - y,w;;:Day5	-1.6	1
ASVM:Day5 - y,w;;:Day5	-7.6	0.99956056
SVM:Day5 - y,w;;:Day5	-1.85	1
S31A:Day5 - y,w;;:Day5	-8.85	0.99593294
ASVM:Day5 - H3.3:Day5	-6	0.99999228
SVM:Day5 - H3.3:Day5	-0.25	1
S31A:Day5 - H3.3:Day5	-7.25	0.99979315
SVM:Day5 - ASVM:Day5	5.75	0.99999871
S31A:Day5 - ASVM:Day5	-1.25	1
S31A:Day5 - SVM:Day5	-7	0.9999527

Appendix Table S10: 2-way ANOVA results of cell cycle durations for RNAi/mutant embryos as shown in Figure EV4B.

Comparison groups	Difference in means	Adjusted p-value
NC11_Slbp-RNAi - NC11_Control	0.69586667	0.99950082
NC12_Slbp-RNAi - NC12_Control	4.4048	3.30E-05
NC13_Slbp-RNAi - NC13_Control	8.49575	2.35E-08
NC11_zelda-RNAi - NC11_Control	0.87616667	0.97411098
NC12_zelda-RNAi - NC12_Control	2.45433333	0.01554046
NC13_zelda-RNAi - NC13_Control	8.03791667	7.69E-14
NC11_Chk1$^{-/-}$ - NC11_Control	0.42016667	0.99997836
NC12_Chk1$^{-/-}$ - NC12_Control	-0.5474091	0.99912207
NC13_Chk1$^{-/-}$ - NC13_Control	-5.05825	1.13E-05
