# Supplementary material for: Local nuclear to cytoplasmic ratio regulates H3.3 incorporation via cell cycle state during zygotic genome activation
Source: EMBO Rep. 2025 Nov 11;26(23):5703–27. doi: 10.1038/s44319-025-00596-1 (PMC12678481; doi:10.1038/s44319-025-00596-1)
Supplement: Supplementary file 8 — Expanded View Figures [file 44319_2025_596_MOESM8_ESM.pdf]

Expanded View Figures

Figure EV1. Tools and controls underlying image quantification of mitotic and interphase Dendra2 and H3.3 export.

(**A**) A 5 kb region containing a single histone gene cluster with one copy of each of the 5 replication-coupled histones, including their promoters, and UTRs as in the endogenous locus in which H3 was N-terminally tagged with the green-to-red photo-switchable fluorophore Dendra2 was inserted at the attp40 site on chromosome 2L (from Shindo and Amodeo, 2019). (**B**) The endogenous H3.3A gene locus was edited to express an N-terminally tagged H3.3-Dendra2 using CRISPR/Cas9 (from Shindo and Amodeo, 2019). (**C**) Total pixel intensities (corresponding to total amounts) on mitotic chromatin for H3-Dendra2 (purple) and H3.3-Dendra2 (green) between NC10-13, normalized to the average individual NC10 values. Chromatin-bound H3 decreases over NC10-13, whereas H3.3 increases. (**D**) Maximum intensity projections of H3-Dendra2 (top) and H3.3-Dendra2 (bottom) on mitotic chromatin from NC10-13. Images are pseudo-colored with nonlinear look-up tables such that purple indicates low intensities and yellow indicates high intensities. H3 intensities fall, and H3.3 intensities rise over the cycles. Scale bar 20 µm. (**E**) The total pixel intensity of individual photoconverted H3.3-Dendra2 nuclei in NC11-13 ($n = 5$). Time is shown relative to nuclear envelope breakdown (NEB). H3.3-Dendra2 intensity remains constant before NEB, indicating that the nuclear export is negligible. The loss of red signal at NEB represents the pool of unbound H3.3 in the nucleus. Each trace represents a single nucleus, each from different embryos. These data were used to plot the unbound H3.3 fraction in Fig. 2E. (**F**) Initial slopes of the nuclear import curves (change in total nuclear intensity over time for the first five timepoints) shown in (2**C**, **D**) for NC11-13. All slopes are normalized to NC11 values. (**G**, **H**) Total pixel intensity of H3-Dendra2 on chromatin (**G**) and interphase nuclei (**H**) with and without continued laser exposure. Two parallel embryos were used to obtain NC10 and NC13 mitotic chromatin with different levels of laser exposure for photobleaching correction (see Methods for details). The data were divided into three regions: "Image area" corresponds to nuclei imaged throughout NC10-13; "outside" corresponds to nuclei outside the image area, but within the imaged embryo; and "parallel embryo" corresponds to nuclei that were only imaged once in NC10 and once in NC13 without continued exposure. Photobleaching was observed to be negligible in experiments quantifying both the mitotic chromatin and interphase nuclear concentration. (Statistical comparisons for (**C**, **E**, **G**, **H**) can be found in Appendix Tables S1, 3–5).

 © The Author(s)

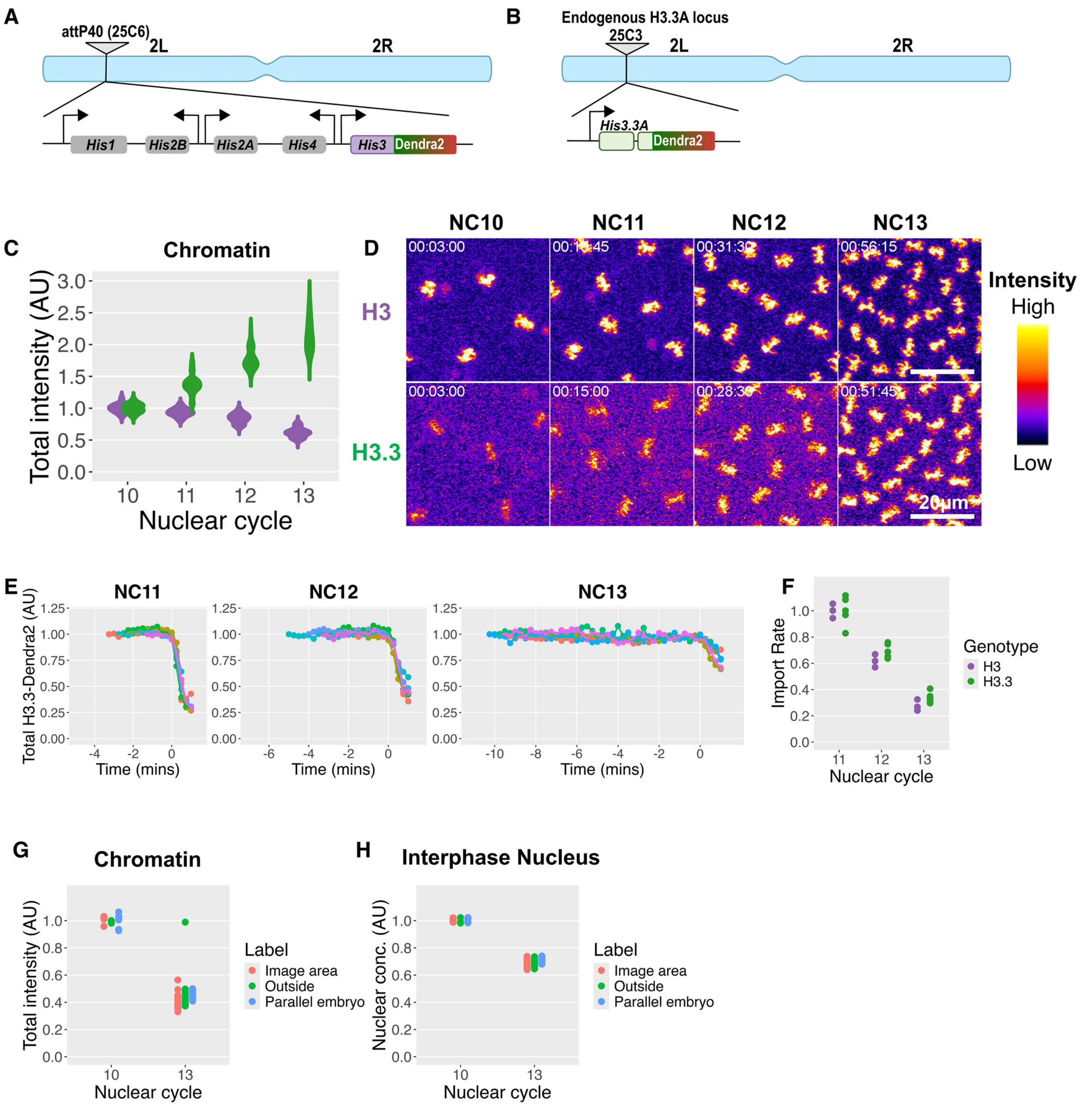
A
attP40 (25C6)
2L
2R
His1
His2B
His2A
His4
His3
Dendra2
B
Endogenous H3.3A locus
25C3
2L
2R
His3.3A
Dendra2
C
Chromatin
Total intensity (AU)
Nuclear cycle
D
NC10
NC11
NC12
NC13
H3
H3.3
Intensity
High
Low
20μm
E
NC11
NC12
NC13
Total H3.3-Dendra2 (AU)
Time (mins)
F
Import Rate
Nuclear cycle
Genotype
H3
H3.3
G
Chromatin
Total intensity (AU)
Nuclear cycle
Label
Image area
Outside
Parallel embryo
H
Interphase Nucleus
Nuclear conc. (AU)
Nuclear cycle
Label
Image area
Outside
Parallel embryo

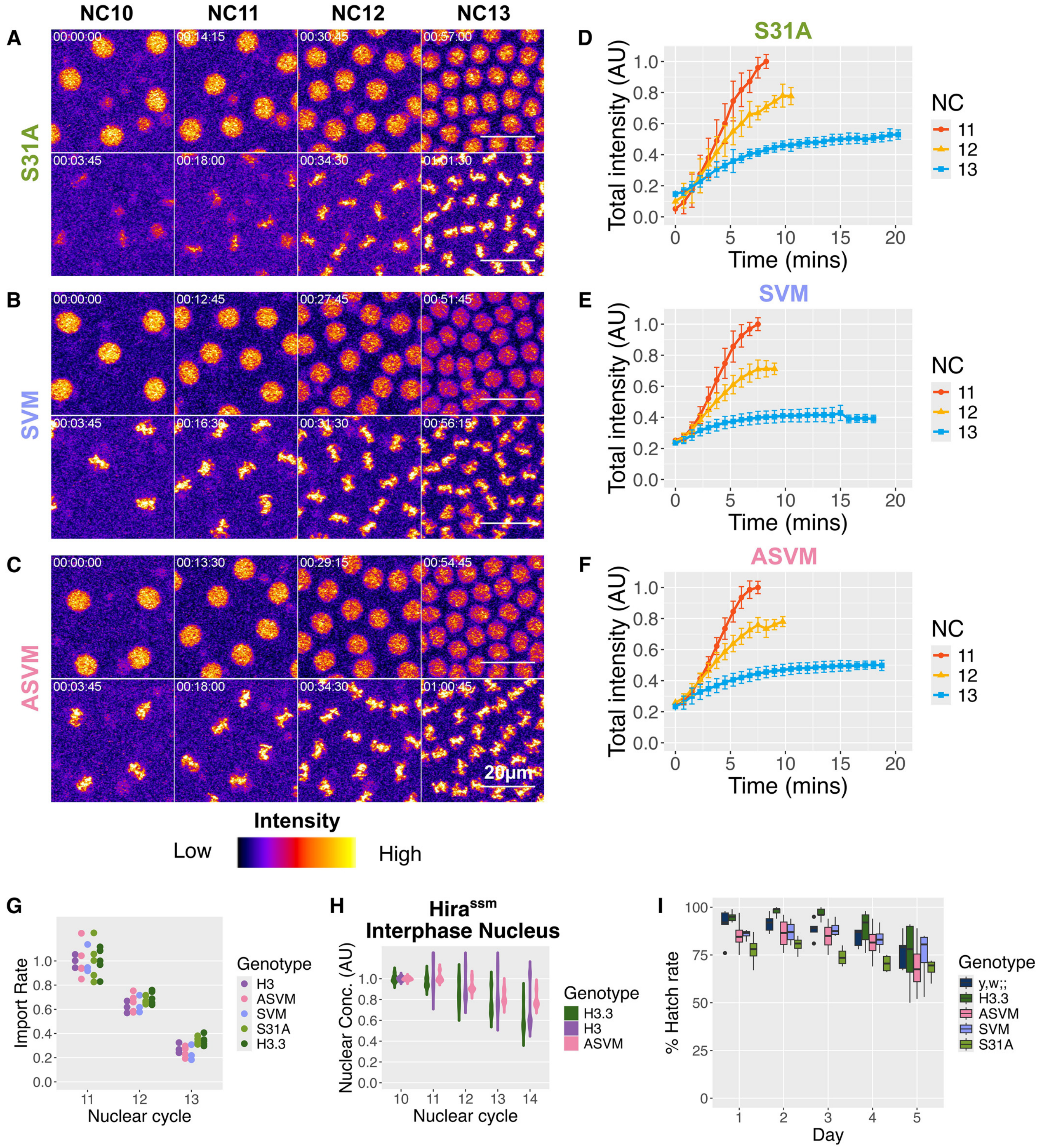
NC10
NC11
NC12
NC13
A
S31A
B
SVM
C
ASVM
20μm
Intensity
Low
High
D
S31A
E
SVM
F
ASVM
Total intensity (AU)
Time (mins)
NC
11
12
13
G
Import Rate
Nuclear cycle
Genotype
H3
ASVM
SVM
S31A
H3.3
H
Hira^ssm
Interphase Nucleus
Nuclear Conc. (AU)
Nuclear cycle
Genotype
H3.3
H3
ASVM
I
% Hatch rate
Day
Genotype
y,w;;
H3.3
ASVM
SVM
S31A

 © The Author(s)

Figure EV2. Representative images of H3/H3.3 chimeras, import curves.

(**A**) Representative maximum intensity projections of H3.3^{S31A}-Dendra2 during interphase and mitosis over NC10-13: interphase nuclei (top) and mitotic chromatin (bottom). Images are pseudo-colored with nonlinear look-up tables such that purple indicates low intensities and yellow indicates high intensities. H3.3^{S31A} behaves similarly to H3.3. (**B**) Representative maximum intensity projections of H3.3^{SVM}-Dendra2 during interphase and mitosis over NC10-13: interphase nuclei (top) and mitotic chromatin (bottom). H3.3^{SVM} behaves similarly to H3. (**C**) Representative maximum intensity projections of H3.3^{ASVM}-Dendra2 during interphase and mitosis over NC10-13: interphase nuclei (top) and mitotic chromatin (bottom). H3.3^{ASVM} behaves similarly to H3. Data from embryos in A-C are quantified in Fig. 3. Scale bar 20 μm. (**D–F**) Total pixel intensities over time for NC11-13 normalized to the maximum NC11 values for H3.3^{S31A}-Dendra2 (**D**), H3.3^{SVM}-Dendra2 (**E**), and H3.3^{ASVM}-Dendra2 (**F**). H3.3^{S31A}-Dendra2 import is similar to H3.3-Dendra2, and only slows after 5 min without plateauing. H3.3^{SVM}-Dendra2 and H3.3^{ASVM}-Dendra2 import in a similar manner to H3-Dendra2 and plateau after 5 min. The solid line represents the mean, and the error bars represent the standard deviation. (**G**) The initial slopes of nuclear import curves of chimeras are shown in (**D–F**) for NC11-13. H3-Dendra2 and H3.3-Dendra2 slopes from S1E are included for reference. All slopes are normalized to NC11 values. (**H**) Average interphase nuclear intensities of H3.3-Dendra2 (green), H3-Dendra2 (purple), and H3.3^{ASVM}-Dendra2 (pink) in Hira^{ssm} embryos 45 s before the NEB in NC10-14, normalized to their average intensities in NC10. Though H3.3 is not incorporated, it is imported into the nucleus, and its concentrations reduce with each cycle. H3 nuclear concentrations also drop with each cycle. However, H3.3^{ASVM} concentrations are relatively more stable over the cycles. ($n = 5$ all chimeras, 3 H3 ssm, 4 H3.3 ssm, and 5 H3.3^{ASVM} ssm embryos.) (**I**) Five-day hatch rates of the H3/H3.3 replacement chimeras compared to control (y,w;;) and H3.3-Dendra2. ($n = 4$ sets of egg-laying cages for each genotype. Statistical comparisons for (**G**, **I**) can be found in Appendix Tables S8, 9).

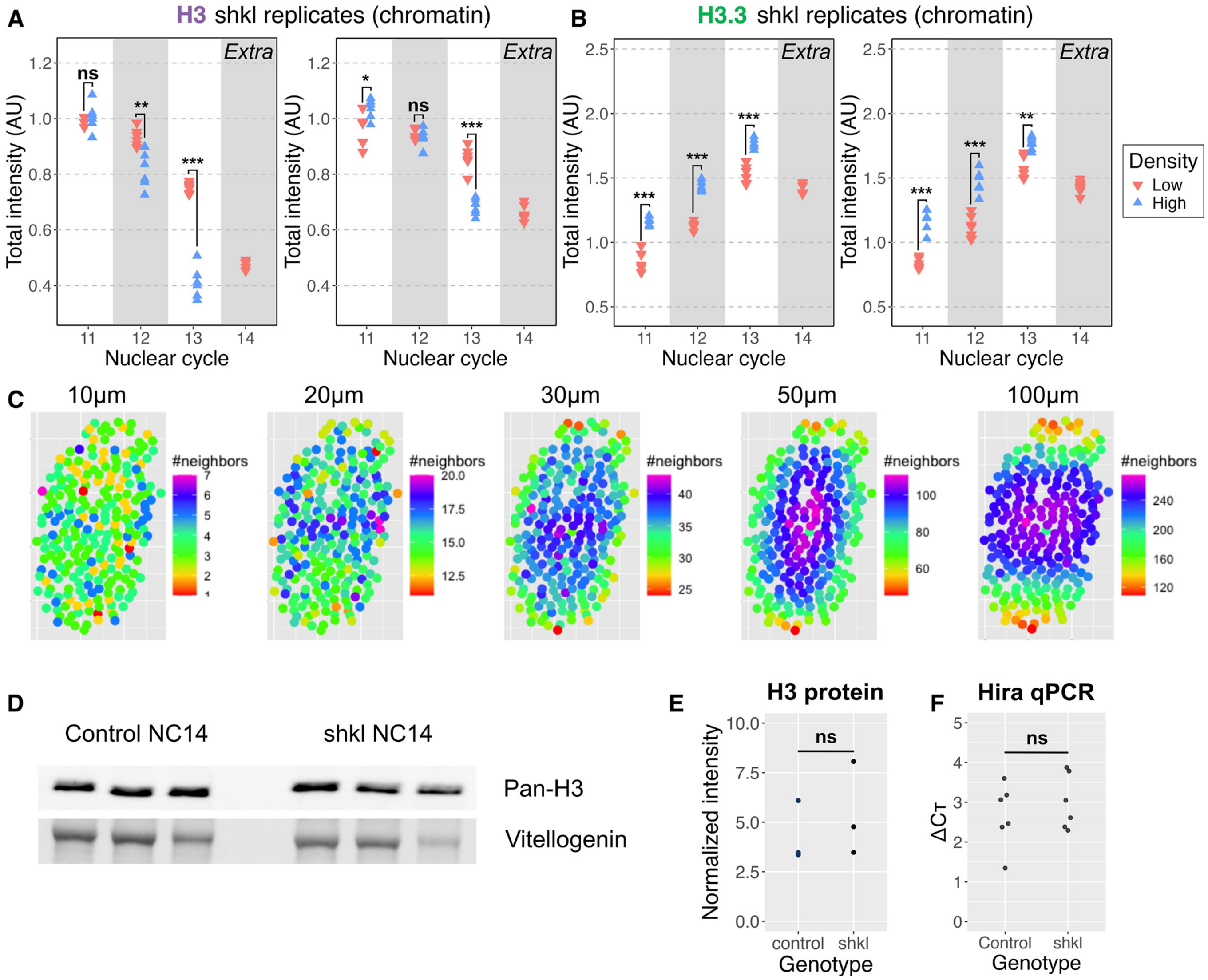

Figure EV3. shkl embryos consistently respond to the N/C ratio without changes in total H3 loading.

(**A**) Replicate shkl embryos of the same genotype as shown in Fig. 4E demonstrate that H3-Dendra2 is consistently retained on mitotic chromatin by nuclei at low-density regions compared to high-density regions within the same cell cycle over NC11-14. (Embryo 1: $p = 0.99$ (NC11), 1.63e-03 (NC12), <e-15 (NC13), Embryo 2: $p = 4.26\text{e-}02$ (NC11), 0.99 (NC12), 2.69e-09 (NC13)). (**B**) Replicate shkl embryos of the same genotype as shown in Fig. 4F demonstrate that H3.3-Dendra2 incorporation is reduced on mitotic chromatin by nuclei at low-density regions compared to high-density regions within the same cell cycle over NC11-14. (Embryo 1: $p = 2.01\text{e-}09$ (NC11), 3.67e-10 (NC12), 5.19e-07 (NC13), Embryo 2: $p = 1.21\text{e-}06$ (NC11), 6.48e-10 (NC12), 1.53e-03 (NC13)) (**C**) Example control embryo in which the radius used to determine the number of neighbors was varied from 10 to 100 μm, as shown. A 20 μm radius was deemed optimal for neighborhood analysis as it enabled us to accurately capture the gradient observed in the shkl embryos while excluding edge effects due to embryo curvature. (**D**) Western blot against a Pan-H3 antibody in control and shkl NC14 embryos. Stainfree signal for Vitellogenin (~45 kDa) is used as a loading control. (**E**) Quantification of (**D**). Pan-H3 levels are not significantly different between control and shkl embryos. (**F**) RT-qPCR results for Hira mRNA in control and shkl NC14 single embryos. Differences in ΔC_T values are not significant. (Statistical significance was determined by one-way/two-way ANOVA, ns= $p > 0.05$, $^*p < 0.05$, $^{**}p < 0.01$, $^{***}p < 0.001$).

 © The Author(s)

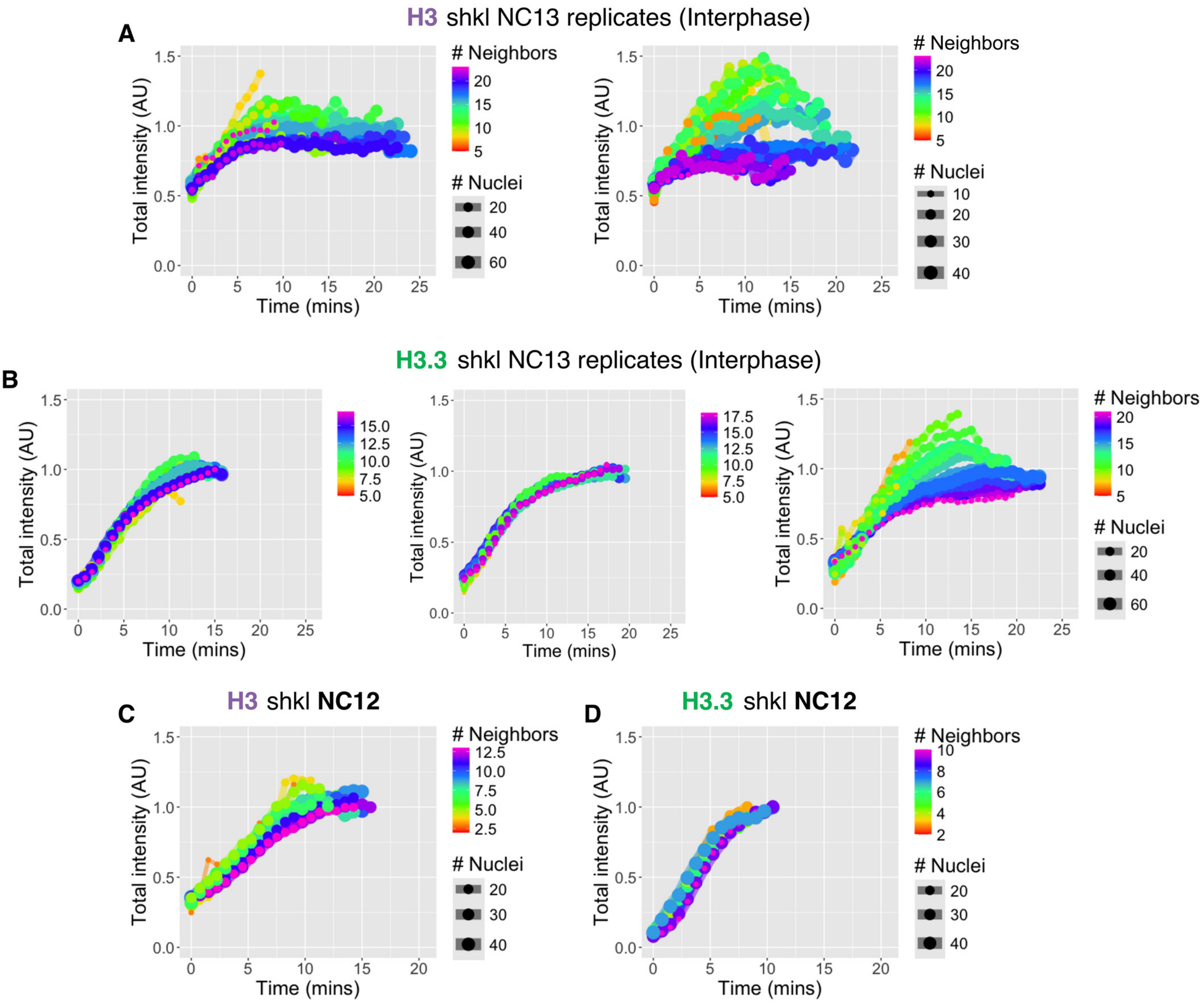

Figure EV4. Replicate shkl embryos demonstrate consistent effects.

(**A**) Replicate shkl embryos of the same genotype as shown in Fig. 5F demonstrate that nuclear import and accumulation of H3 inversely correlate with the number of neighbors surrounding a given nucleus, suggesting H3 nuclear import is N/C ratio sensitive. (**B**) Replicate shkl embryos of the same genotype as shown in Fig. 5G demonstrate that nuclear import and accumulation of H3.3 is less N/C ratio sensitive than H3 in most cases. (**C**, **D**) Total intensities over time for H3-Dendra2 (**C**) and H3.3-Dendra2 (**D**) in NC12 shkl embryos indicate that the trends observed in NC13 begin in NC12, though to a lesser extent. These data were taken from the same embryo that was used in Fig. 5F,G, respectively.

© The Author(s)

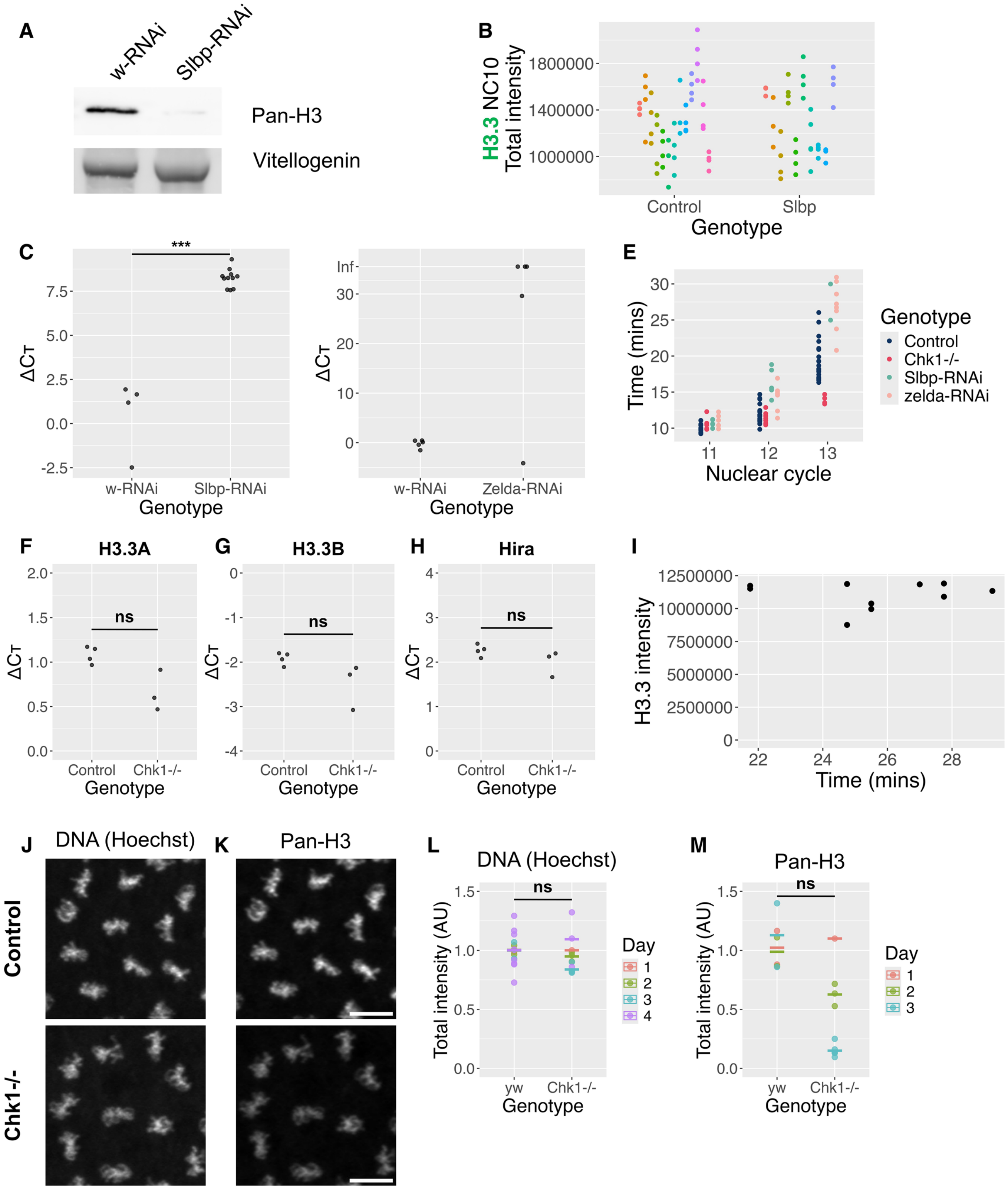
A
w-RNAi
Slbp-RNAi
Pan-H3
Vitellogenin
B
H3.3 NC10
Total intensity
1800000
1400000
1000000
Control
Slbp
Genotype
C
***
ΔCT
7.5
5.0
2.5
0.0
-2.5
w-RNAi
Slbp-RNAi
Genotype
Inf
30
20
10
0
w-RNAi
Zelda-RNAi
Genotype
E
Time (mins)
30
25
20
15
10
11
12
13
Nuclear cycle
Genotype
Control
Chk1-/-
Slbp-RNAi
zelda-RNAi
F
H3.3A
ns
2.0
1.5
1.0
0.5
0.0
Control
Chk1-/-
Genotype
G
H3.3B
ns
0
-1
-2
-3
-4
Control
Chk1-/-
Genotype
H
Hira
ns
4
3
2
1
0
Control
Chk1-/-
Genotype
I
H3.3 intensity
12500000
10000000
7500000
5000000
2500000
0
22
24
26
28
Time (mins)
J
DNA (Hoechst)
K
Pan-H3
Control
Chk1-/-
L
DNA (Hoechst)
ns
Total intensity (AU)
1.5
1.0
0.5
0.0
yw
Chk1-/-
Genotype
Day
1
2
3
4
M
Pan-H3
ns
Total intensity (AU)
1.5
1.0
0.5
0.0
yw
Chk1-/-
Genotype
Day
1
2
3

© The Author(s)

Figure EV5. Quantification of histone composition and cell cycle times in RNAi and mutant embryos.

(**A**) Western blot against a Pan-H3 antibody in w-RNAi (control) and Slbp-RNAi embryos after a 1 h collection. Stainfree signal for Vitellogenin (~45 kDa) is used as a loading control. Slbp-RNAi causes H3 knockdown. (**B**) H3.3 incorporation in NC10 mitotic chromatin in w-RNAi (control) and severely affected Slbp embryos, which do not survive past NC11 due to mitotic defects. H3.3 levels are comparable between w-RNAi (control) and Slbp embryos. Different colors represent nuclei from different embryos. (**C**) RT-qPCR results for Slbp mRNA in w-RNAi (control) and Slbp-RNAi single embryos from a 2 h collection. Slbp mRNA is significantly reduced ($p = 2.04e\text{-}08$) in RNAi embryos. (**D**) RT-qPCR results for Zelda mRNA in w-RNAi (control) and Zelda-RNAi single embryos from a 1 h collection. Zelda mRNA is dramatically reduced in RNAi embryos, often undetectable. (**E**) Cell cycle durations of control (a mix of y,w and w-RNAi) and RNAi/mutant embryos used in Fig. 6. Note that cell cycles are shortened in Chk1$^{-/-}$ (*H3.3-Dendra2, grp^{1}*) embryos and lengthened in Slbp-RNAi and Zelda-RNAi embryos. (**F**–**H**) RT-qPCR results for H3.3 A (**F**), H3.3B (**G**), and Hira (**H**) mRNA in control (H3.3-Dendra2) and Chk1$^{-/-}$ (H3.3-Dendra2, grp^{1}) mutant NC12 single embryos. Differences in ΔC_T values are not significant. (**I**) H3.3 chromatin incorporation versus NC13 duration in control (H3.3-Dendra2) embryos. No correlation is observed between NC13 cell cycle duration and H3.3 incorporation. (**J**, **K**) DNA (**J**) and Pan-H3 (**K**) staining in control (y,w) and Chk1$^{-/-}$ mutant NC12 embryos. Scale bar 10 μm. (**L**) Quantification of J. DNA amounts are unchanged in the Chk1$^{-/-}$ mutant. (**M**) Quantification of K. Pan-H3 stain is more variable in the Chk1$^{-/-}$ mutant. Each point indicates the average amount in a single embryo, and solid lines indicate average amounts from all the embryos of a single day. Day averages were used to perform one-way ANOVA significance tests. ($n \geq 3$ embryos. Statistical significance was determined by one-way/two-way ANOVA, ns= $p > .05$, *$p < .05$, **$p < 0.01$, ***$p < 0.001$. Statistical comparisons for (**E**) can be found in the Appendix Table S10).

© The Author(s)
